# Pinna synthetic mold for otoplasty techniques application^☆^

**DOI:** 10.1016/j.bjorl.2017.01.004

**Published:** 2017-02-14

**Authors:** Mariah Guieiro Alves dos Reis, Ricardo Guimarães Marim, Luis Ricardo Martinhão Souto

**Affiliations:** a Faculdade de Medicina de Marília, Departamento de Otorrinolaringologia, Marília, SP, Brazil; b Faculdade de Medicina de Marília, Departamento de Otorrinolaringologia, Divisão de Otorrinolaringologia e Cirurgia Plástica, Marília, SP, Brazil

**Keywords:** Prominent ears, Otoplasty, Surgical techniques, Orelhas proeminentes, Otoplastia, Técnicas cirúrgicas

## Abstract

**Introduction:**

The ear deformity Tanzer type V, also known as prominent ears, is the most common genetic defect of the pinna. The surgery designed for its correction is known as otoplasty. This esthetic surgery can be performed using different techniques, which requires great skill of its operator.

**Objective:**

The purpose of this work is the development of a new tool for otoplasty techniques training, aimed on the possibility to minimize errors during the otoplasty.

**Methods:**

Synthetic molds of the external ear from patients with Tanzer type V deformity were made, using silicone material and rayon.

**Results:**

The main procedures of otoplasty could be performed in the molds made of silicone and rayon with a good esthetic result.

**Conclusion:**

The elaborated molds had identical size and shape of a human ear and could be positioned in the same shape of the patient ears. Thus, the synthetic molds were presented as promising simulation tools for the training and surgical enhancement of otoplasty, especially for doctors beginners.

## Resumo

**Introdução:**

A deformidade da orelha tipo V de Tanzer, também conhecida como orelhas proeminentes, é o defeito genético mais comum da aurícula. A cirurgia criada para sua correção é conhecida como otoplastia. Essa cirurgia estética pode ser realizada utilizando-se diferentes técnicas, o que requer grande habilidade de seu operador.

**Objetivo:**

O objetivo desse trabalho é o desenvolvimento de uma nova ferramenta para o treinamento de técnicas de otoplastia, com o objetivo de minimizar erros durante a otoplastia.

**Método:**

Foram realizados moldes sintéticos da orelha externa de pacientes com deformidade tipo V de Tanzer, utilizando material de silicone e rayon.

**Resultados:**

Os principais procedimentos de Otoplastia foram realizados nos moldes feitos de silicone e rayon com um bom resultado estético.

**Conclusão:**

Os moldes elaborados tinham tamanho e forma idênticos aos de uma orelha humana e puderam ser posicionados no mesmo formato das orelhas dos pacientes. Assim, os moldes sintéticos foram apresentados como ferramentas de simulação promissoras para o treinamento e aperfeiçoamento cirúrgico da otoplastia, especialmente para médicos iniciantes.

## Introduction

The ear deformity Tanzer type V, also known as prominent ears or “floppy”, is the most common genetic defect of the pinna. It is mainly caused by the anti-helix blemish, sharp protrusion and shell development, or a mixture of both.^1^

Not earlier than in the end of the 19th century, reports on surgical techniques used to pin down protruding ears for cosmetic reasons were published. Dieffenbach was among the first when, in 1845, he described his technique of otoplasty to correct a posttraumatic prominent auricle in a patient.^2^

In the literature, there are studies using animal models to employ surgical techniques of otoplasty, however, there is still no description of synthetic models for this purpose.

Nonlive models are used for a student to acquire a set of basic surgical skills, especially in the early stages of learning. Thus, the number of animals used for training purposes decreases and it is developed the students’ confidence to work with alive tissues.^3^

In this work we applied Stenstrom, Mustardé and Furnas techniques to ear deformity correction, in synthetic ear molds made of silicone material.

## Methods

### General objective

Development of an external ear mold with Tanzer type V ear deformity for otoplasty surgery training.

### Specific objectives

Development of external ear molds of patients with Tanzer type V ear deformity (protruding ears).

Application of otoplasty surgical technique in elaborated molds.

### Reasons

In the literature there are more than two hundred procedures for the treatment of “protruding ears”.^4^ Excessive ear prominence can be the result of failure of scapha folding, conchal hypertrophy, conchal malposition, or a combination of these deformities. Management of this problem is based on the accurate diagnosis of the deformity and a thorough understanding of the basic techniques that address them.^5^

Surgical correction of ear prominence is one of the few purely esthetic surgeries whose appointment is widely accepted not only in adults but also in children and teenagers.^6^, ^7^, ^8^ The main goal of the treatment is to achieve an acceptable placement of the ear, symmetry and fitness, contributing to the satisfaction of patients and their families.^9^

The otoplasty techniques are easy to learn and are very useful in medical residents training.^10^ It is noted, therefore, the importance of other means for otoplasty techniques training while the medical resident learning, at the expense of directly technique application on the patient.

In this sense, this paper proposes the creation of external ear molds made with silicone, for the otoplasty techniques application, in order to be used as a viable tool in the training and surgical skills improvement of doctors’ beginners.

### Ethical aspects

This study is in accordance with 196/96 Resolution of the National Council of Ministry of Health, which regulates research involving human subjects. It is noteworthy also that the molds only raced after the informed consent signing of patients involved (Appendix A).

### Patient selection

Five patients treated at the Otorhinolaryngology Department, who presented shell and anti-helix defects in the outer ear were randomly chosen. These patients were asked to perform the molding of their pinna prior to completion of otoplasty surgery.

### Molds manufacture

Ten molds of external ear were performed – five left ear molds and five templates of the contralateral ear of five patients who were about to realize an otoplasty surgery at the Otorhinolaryngology Department.

The molds were made with silicone-based media -A.ZOFT, Oticon^®^^11^ routinely used in the individual sound amplification devices (HA) pre-molding^12^ at the Otorhinolaryngology Department. In the HA preform molding it is used the technique described by Pirzansk (1997) and Oliveira (1997), which serve for printing a shell ear and an External Auditory Meatus (EAM).^13^, ^14^ This impression (preform) is the basis for the further production of an acrylic or a silicone mold that makes up the hearing aids prepared by prosthetic specialized companies.

**Figure 1:**
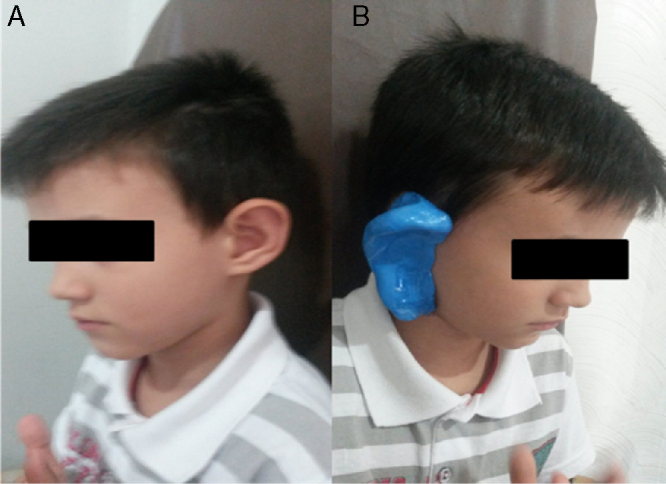
(A) A patient with a V Tanzer ear deformity. (B) Pre-molding performed on the patient's right ear.

**Figure 2:**
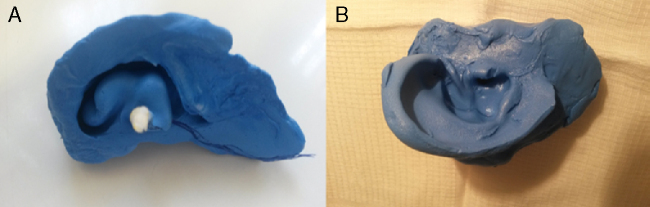
(A) Counter-mold made of silicone from the ear of a patient with protruding ears. (B) Pinna right template, made with the application of resin on the counter-mold.

**Figure 3:**
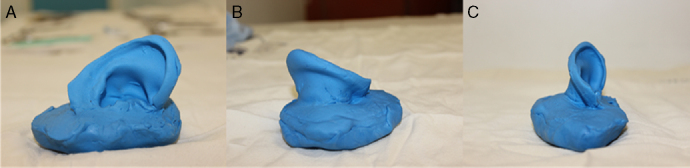
Ear mold made from the countermold in previous view (A), posterior view (B) and lower view (C).

In the pre-molding, firstly a cotton plug tied with a long and tough wire is placed at the end of the second EAM curve with the aid of a flashlight with a special acrylic tip for this purpose. Then, a homogeneous mixture of the modeling mass (Silicone Base Catalyst Component) is inserted in the EAM with the help of an appropriate syringe. After this step, the shell, the tragus and the helix are filled, by compressing the mass delicately with the fingers in these areas. To create a mold of the external ear, an extension of the conventional molding was performed, in which additionally we coated the anti-tragus, lobe and the posterior region of the pinna. Thus, it was obtained an entire printing of the auricle and MAE (Fig. 1). After drying for 5–10 min, the resin was gently detached from the ear of the patient flag and gave the counter-mold of the external ear (Fig. 2). This was coated with petroleum jelly and then filled with the same printing material. After a further drying period, the mold was removed from the counter-mold and led to a replica of the patient's external ear (Figure 2, Figure 3).

In each of the ten molds surgical otoplasty procedures were applied. In this work we applied a combination of Mustardé, Stenström and Furnas otoplasty techniques.

**Figure 4:**
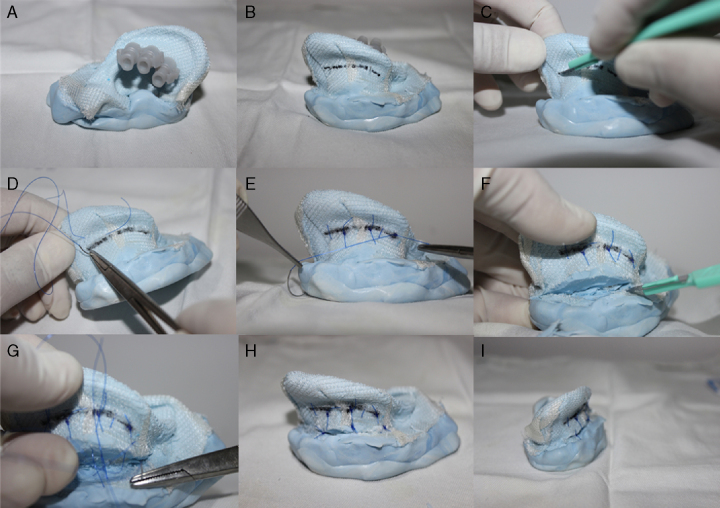
(A) Otoplasty technique performed in the mold covered with rayon. The anti-helix dial with needles. (B) Extended anti-helix marking with permanent marker pen. (C) Incision not transfixing at the anti-helix posterior region. (D) “U” suture at the antihelix posterior region with Prolene^®^ 4.0 thread. (E) Anti-helix established without breaking the resin or the rayon. (F) Incision in the back of the shell with number 15 scalpel blade. (G) Not transfixing suture of the shell posterior region in the mastoid area. Back View (H) and upper view (I) of the mold at the end of otoplasty procedure.

**Figure 5:**
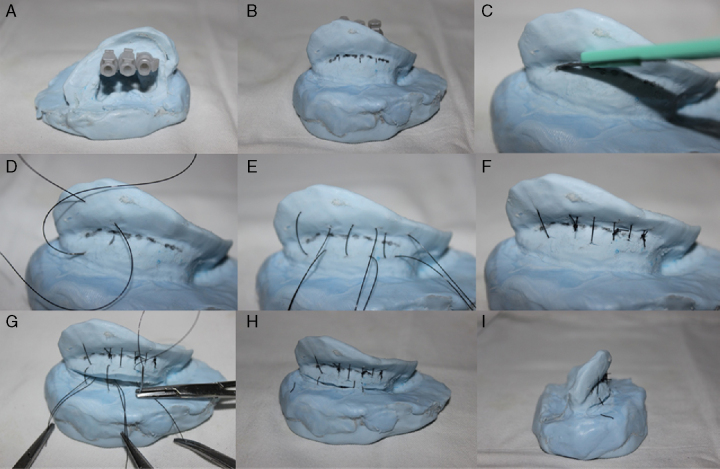
Otoplasty performed in the mold with an inner lining of rayon. The anti-helix dial with needles (A). The anti-helix dial extension with permanent marker pen (B). Incision not transfixing the posterior region of the anti-helix (C). “U” suture of the anti-helix posterior region with 4.0 Prolene (D and E). Anti-helix established without breaking the resin or rayon (F). Incision in the posterior region of the shell with a scalpel number 15 and a non-transfixing shell suture in the mastoid region (G). Back View (H) and upper view (I) of the mold at the end of the otoplasty procedure.

For the anti-helix correction was first carried out three reference points marked with the mold transfixation by 26G needles (0.45 × 13): one in the anti-helix bifurcation region, one in the middle region and the last at the root of the anti-helix (Figure 4, Figure 5). Guided by these needles, the antihelix demarcation in the posterior region of the pavilion with a marker is made (Figure 4, Figure 5).

**Figure 6:**
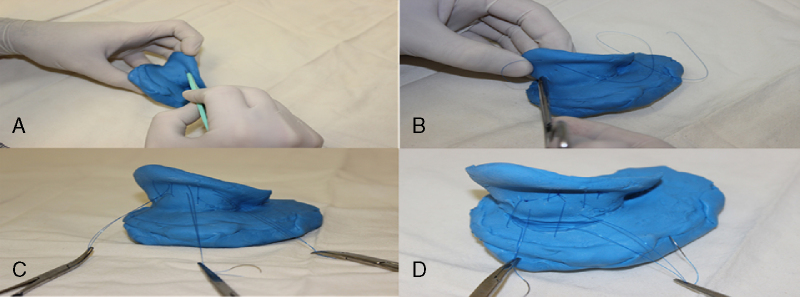
(A) Wear of the anti-helix posterior region with a scalpel blade number 15. (B) and (C) suture in a “U” format in the posterior region of the anti-helix. (D) Suture of the shell posterior region in the mastoid area.

After the withdrawal of the needles a wear of the demarcated area with scalpel blade 15 was made (Figure 4, Figure 5, Figure 6). Then sutures in a “U” format at three points of the marked area were made: one in the bifurcation region of the anti-helix, one in the middle region and the other in the anti-helix root, so as to bend this area and create a neo-anti-helix (Figure 4, Figure 5, Figure 6).

For the shell correction, an incision with scalpel blade 15 was made in the retroauricular sulcus (Fig. 4F). Then, there were realized three sutures in the “U” format, each beginning at the shell region in a proximal-distal direction and ending in a distal-proximal direction in the region of the base (mastoid) of the mold, permitting the shell rotation (Figure 5, Figure 6). Thus, the angle between the concha and the mastoid region was reduced.

For not having a coating that simulated human skin, ear molds do not allowed the realization of all otoplasty surgical steps. In this way, it was not possible the local anesthetic infiltration, surgical removal of skin excess, dissection of the subcutaneous tissue or excision of the retroauricular muscle. The development of a neo-anti-helix and the repositioning of the shell were the surgical steps performed.

The silicone molds showed similar elasticity and resistance to the human ear cartilage. The elasticity allowed the anti-helix and shell mobilization and repositioning. However, just like an ear cartilage, silicone has low resistance and it was broken when sutured. In the human ear is the auricular cartilage perichondrium that ensures the support of the suture and not the cartilage.

The molds made solely with silicone served to apply the main stages of otoplasty, but there was a material rupture both with the use of Prolipropilene 4.0 wires or Nylon 4.0, then, it was not possible to keep the molds in the desired position after the surgical procedures. Thus, it was possible to train otoplasty techniques along these molds, but it was not possible to sustain them in the new position (Fig. 6).

**Figure 7:**
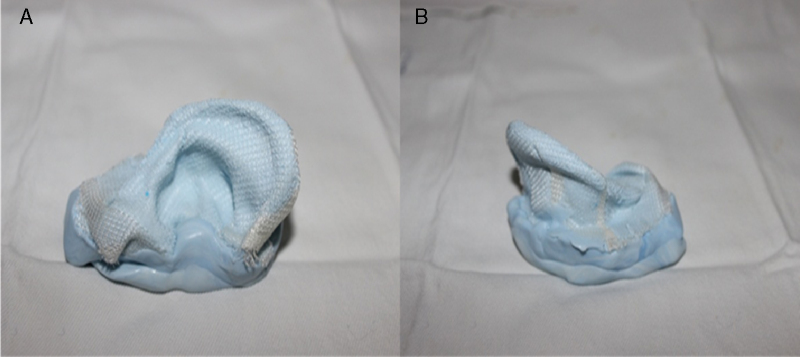
Rayon applications externally in the ear mold to increase its strength.

In an attempt to simulate the perichondrial tissue, new silicone molds were made and received a layer of rayon. First the mold was coated with a rayon fabric (Fig. 7). In this new format the molds had sufficient elasticity and strength for otoplasty techniques application and were not broken by sutures (Fig. 4).

Otherwise, it was added rayon inside the mold, which also ensured adequate elasticity and strength for the otoplasty procedure without the material rupture (Fig. 5).

## Results

The main procedures performed in the molds were the anti-helix construction and the shell rotation, which are the basic steps of otoplasty. These steps allow the correction of the two determinant amendments of the protruding ears: total or partial absence of the anti-helix and shell protrusion.

The molds elaborated with silicone had identical size and shape of a human ear and could be positioned in the same shape of the patient ears. With a layer of rayon, the molds showed a better elasticity and allowed the good practice of otoplasty steps.

## Discussion

An auricular prominence is characterized by the angle greater than 40° between the ear and the mastoid plane, or a greater distance than 21 mm between these two structures.^1^, ^2^, ^3^, ^4^, ^5^, ^6^, ^7^, ^8^, ^9^, ^10^, ^11^, ^12^, ^13^, ^14^, ^15^ The surgical procedure for this deformity correction is called otoplasty.

This surgery can be performed with three main steps: scraping (wear), excision (removal) and fixation of the auricular cartilage by sutures.^4^, ^5^, ^6^, ^7^, ^8^, ^9^, ^10^, ^11^, ^12^, ^13^, ^14^, ^15^ There are, however, many surgical techniques used in this procedure, highlighting the technique developed by Mustardé, Stenstrom and Furnas (1968)^16^, ^17^, ^18^ in which the ear cartilage is sutured, transfixing it to the previous perichondrium, in the mastoid fascia, with unabsorbed thread. Detailing the techniques separately, it is known that Mustardé was based on the posterior access of the antihelix and the suture of the region with non-absorbable thread, comprising the above perichondrium without transfix the skin^16^; Stenstrom (1963) performed scraping of the anti-helix area to create softer outline;^17^ and Furnas (1968) sutured the ear cartilage transfixing it to the previous perichondrium, in the mastoid fascia, with non-absorbable thread.^18^

Regardless of the technique to be employed, the otoplasty is a surgical procedure that requires great skill of the surgeon in performing all its steps. Therefore, it is important to note that training and simulation are extremely important to prepare the medical residents in surgery to perform well the procedures in the initial learning curve phase,^19^ especially in the implementation of cosmetic procedures such as the correction of ear deformities.

It is known that the modern concept of simulation came in the aviation area with the development of the first flight simulator by the American engineer Edwin A. Link. In health, however, the use of simulators is still relatively recent, but studies show that they have gained ground as important training methods used to minimize the risk in the activities and increase the safety of processes.^20^

This study aims to demonstrate that this synthetic ear mold allows the training of otoplasty techniques, since it has proper anatomical reliance and provides approximate sense of surgery. Despite of the impossibility to perform some initial steps of the surgical technique, such as the local anesthetic infiltration, the molds were considered satisfactory by the surgeons and assistants of the Otorhinolaryngology Department who analyzed and handled them.

## Conclusion

Synthetic auricular molds made only with silicone allowed the application of otoplasty techniques, but suffered disruption after the procedures. In contrast, the molds made of silicone and rayon provided sufficient elasticity and strength without breaking, ensuring a satisfactory end result and presenting themselves as suitable simulation tools for the training and surgical enhancement of otoplasty, especially for doctors beginners. The surgical techniques performed in this otologic simulator does not replace the “alive” one, however, the elaborated molds can be presented as a cheap and promising alternative for the practical teaching of the ear esthetic surgery, corroborating the minimization of errors and medical malpractice during the otoplasty surgery.

## Conflicts of interest

The authors declare no conflicts of interest.

## Footnotes

Appendix A Supplementary data associated with this article can be found, in the online version, at doi:10.1016/j.bjorl.2017.01.004.
