# Supplementary material for: Pinna synthetic mold for otoplasty techniques application
Source: Braz J Otorhinolaryngol. 2017 Feb 14;84(2):159–65. doi: 10.1016/j.bjorl.2017.01.004 (PMC9449240; doi:10.1016/j.bjorl.2017.01.004)
Supplement: Supplementary file 1 [file mmc1.pdf]

APPENDIX A

INFORMED CONSENT

PINNA SYNTHETIC MOLD FOR OTOPLASTY TECHNIQUES APPLICATION

This work called " **PINNA SYNTHETIC MOLD FOR OTOPLASTY TECHNIQUES APPLICATION** " is carried out in accordance with the Ethical Standards and has as main objective the construction of a tool (a mold) to facilitate the accomplishment, by beginning doctors, of a surgery Cosmetic (esthetics) of the ear.

In one part of the ear, we have the outer ear, with the pinna (outside). This is where we intend to build our mold, using a soft modeling mass normally used to make hearing aids in patients with hearing difficulties.

Thus, the molds will be made from the molding of your outer ear with a malleable mass. The application of the mass to model the ear is made only from the outside, with the help of a plastic syringe. After molding the ear, we will wait for 1 to 2 minutes for the mass to dry on it, and we will remove the mold. After molding you will be excused from participation. These steps in shaping your ear will pose no risk to your health. We also inform you that the modeling masses used are dermatologically tested by specialists before use.

Each mold will serve for the training of a cosmetic ear surgery, called Otoplasty. With the use of these templates we seek to improve our learning as medical surgeons.

We declare that your participation in this study is voluntary and if you choose not to participate or intend to give up continuing at any time, you will have absolute freedom to do so.

In publishing the results of this work, your identity will be kept in the strictest secrecy. In addition, any information that may identify you will be omitted. Even without the direct benefits of participating, you will be indirectly contributing to the learning of many beginning doctors. We also declare that there will be no refund of money, since with participation in the survey you will not have any expenses.

You will keep a copy of this Term of Consent.

I, ____ register number__________________________, was properly informed regarding the research project " **PINNA SYNTHETIC MOLD FOR OTOPLASTY TECHNIQUES APPLICATION** " and I agree to participate.

Date and Local: .... 2017.

________________________________ ____________________________

Participant's Signature Researcher´s Signature
